# Alcohol Use and Related Problems Among Ethnic Minorities in the United States

**Published:** 2003

**Authors:** Frank H. Galvan, Raul Caetano

**Affiliations:** Frank H. Galvan, Ph.D., L.C.S.W., is a researcher at the Collaborative Alcohol Research Center and an assistant professor at the Charles R. Drew University of Medicine and Science, Los Angeles, California. Raul Caetano, M.D., Ph.D., is a professor and dean of the Dallas Regional Campus, University of Texas Houston School of Public Health, Dallas, Texas

**Keywords:** ethnic differences, minority group, cultural patterns of drinking, prevalence, AODR (alcohol and other drug related) mortality, AOD associated consequences, sociocultural aspects of AOD use, cultural sensitivity, treatment outcome, Hispanic, African American, Native American, Asian American

## Abstract

Alcohol use patterns and the prevalence of alcohol-related problems vary among ethnic groups. Among the elements thought to account for these ethnic differences are social or cultural factors such as drinking norms and attitudes and, in some cases, genetic factors. Understanding ethnic differences in alcohol use patterns and the factors that influence alcohol use can help guide the development of culturally appropriate alcoholism treatment and prevention programs.

Ethnic minorities currently make up about 29 percent of the U.S. population (U.S. Census Bureau 2001).^1^ This article reviews the main research findings on the alcohol consumption patterns and related problems of the four main ethnic minority groups in the United States: Hispanics, Blacks, Asian Americans, and Native Americans. Comparison data are often given for Whites, who make up the majority group in the United States. An understanding of the similarities and differences in alcohol consumption that exist among these ethnic groups and the differences that distinguish them from Whites can guide the development of alcoholism prevention and treatment programs to meet the needs of members of these groups. Although recent alcohol research has emphasized the heterogeneity that exists within each ethnic group (Caetano et al. 1998), detailed examination of within-group differences is beyond the scope of this article.

## Alcohol Consumption Patterns Among U.S. Minority Groups

Patterns of alcohol consumption have been found to vary across ethnic groups. Surveys of nationally representative samples of people age 18 and older conducted in 1984 and 1995 found that the rates of alcohol abstention remained stable among White men but increased among Black and Hispanic men (see table 1) (Caetano and Clark 1998*a*).

This study also found that frequent heavy drinking (defined as drinking five or more drinks at a sitting at least once a week^2^) decreased among White men but remained stable among Black and Hispanic men.^3^ Among women, abstention rates increased among all three groups, with the highest increases occurring among Blacks and Hispanics compared with Whites. Women’s rates of frequent heavy drinking mirrored those of men, with a decrease in heavy drinking among White women and stability of rates for Black and Hispanic women.

Several factors were associated with the likelihood of current frequent heavy drinking for the three groups (Caetano and Clark 1998*a*). Among White men, risk factors for frequent heavy drinking were lower educational attainment and being separated, divorced, or never married. Factors associated with a lower likelihood of frequent heavy drinking for White men were being older than 50 years of age and reporting that religion was important in their lives. No specific risk factors were found for Black men; protective factors included being retired and defining religion as important in their lives. Among Hispanic men, unemployment was the only identified risk factor.

Among White women, never having married was a risk factor for frequent heavy drinking, and being older than 50 was a protective factor. Among Black women, lower income and unemployment were risk factors. Black women between the ages of 50 and 59 were less likely to report frequent heavy drinking than those between 18 and 29. A risk factor for Hispanic women was unemployment; protective factors included older age, retirement, and defining religion as important in their lives.

Data from the 1992 National Longitudinal Alcohol Epidemiologic Survey (NLAES) found drinking patterns for Native Americans to be similar to those of Blacks and Hispanics (specifically, Mexican Americans) (Dawson 1998). Compared with Whites, Native Americans are less likely to drink—that is, a greater percentage of the population abstains—but they consume more alcohol when they do drink, as is the case for both Blacks and Mexican Americans.

Heterogeneity in drinking patterns is also found among different nationalities within specific ethnic groups (Dawson 1998). Blacks whose ancestry is Caribbean consume less alcohol compared with Blacks in general. Hispanic Americans of Central American, South American, or Caribbean ancestry consume less alcohol than Hispanics in general (including Hispanics of Mexican or Mexican American ancestries). Among Asians, Japanese Americans consume more alcohol than Asian Americans of other national origins (Dawson 1998). Heterogeneity in drinking patterns also varies by place of birth. For example, Asians and Pacific Islanders born in the United States have lower alcohol abstention rates than those born elsewhere (Makimoto 1998).

Differences in alcohol consumption are also found among Native Americans. Those living on reservations drink less frequently than Native Americans living in off-reservation towns, but reservation dwellers may engage in binge drinking (drinking five or more drinks per day) more frequently and consume more alcohol per occasion when they do drink (May and Gossage 2001).

## Alcohol-Related Problems

Excessive alcohol consumption can cause social problems such as divorce or job loss and legal problems such as driving under the influence, as well as alcohol abuse and dependence. Surveys conducted in 1984 and 1995 using national probability samples of people age 18 and older found that the proportions of White and Black men reporting one, two, or three or more alcohol-related problems (such as withdrawal, tolerance, accidents, problems with the police, and problems with a spouse) were relatively stable from one time to the next (see table 2) (Caetano and Clark 1998*b*). However, the proportion of Hispanic men reporting three or more problems almost doubled between the two time periods (increasing from 9 percent to 16 percent). Among women of all three ethnic groups, problem prevalence was relatively stable across the two time periods.

Several factors were associated with the likelihood of having alcohol-related problems (Caetano and Clark 1998*b*). For both genders and all three groups, older people were less likely to report having alcohol-related problems than younger people. For all groups, the amount of alcohol a person consumed per week was positively associated with the likelihood of having alcohol-related problems. Separated, divorced, or never married White men and women had a higher risk of alcohol-related problems than those who were married. Among Black men, being widowed was associated with a lower risk for alcohol-related problems compared with married men. Among Hispanic men, the unemployed had a higher likelihood of alcohol-related problems than those who were employed, and Hispanic men with incomes of $40,000 or more had a lower likelihood of reporting alcohol-related problems than those whose incomes were less than $10,000.

Among Black women, homemakers or unemployed women had a higher likelihood of having alcohol-related problems than employed women. Among Hispanic women, those who were retired had a lower likelihood of having such problems than those who were employed.

Variation in alcohol-related problems has also been found to exist within specific ethnic groups. For example, among Hispanics, Mexican Americans have been found to have more alcohol-related problems than those of Cuban and Puerto Rican origins (Caetano et al. 1998).

Information about the prevalence of the specific problems of alcohol abuse and dependence also comes from the National Comorbidity Survey (NCS), a nationally representative household survey of people ages 15 to 54, which used diagnostic interviews to estimate the prevalences of alcohol and other drug abuse disorders (Kessler et al. 1994). This study found that Blacks have significantly lower rates of substance use disorders in general than Whites. However, the NCS did not find a difference between Hispanics and Whites in the rate of alcohol use disorders. In contrast, the Epidemiologic Catchment Area Study, a similar study that reported survey data from the general population as well as an institutionalized population (Helzer et al. 1991), found that Hispanics have higher rates of alcohol use disorders compared with Whites. Data from the NLAES reveal that Whites are more likely than Blacks to develop alcohol dependence, but no more likely than Hispanics (Grant 1997). In addition, Blacks and Hispanics are more likely than Whites to continue their alcohol dependence once it begins.

Heterogeneity in alcohol abuse and dependence also exists within groups with the same national origins. For example, among people of Mexican origin in the United States, Mexican Indians have higher rates of lifetime alcohol abuse or dependence than non-Indian Mexicans (Alderete et al. 2000). However, these differences are not significant after the effects of other sociodemographic factors are taken into account.

Heterogeneity in alcohol-related problems also varies by place of birth. For example, U.S.-born Mexican American women have higher rates of alcohol dependence than Mexican American women born outside the United States (Caetano et al. 1998).

Research has also compared the prevalence of specific alcohol-related problems, such as drinking and driving, for different ethnic groups. A national probability study conducted in 1995 revealed that White and Hispanic men had higher rates of driving after drinking enough “to be in trouble if stopped by the police” in the previous 12 months (22 percent and 21 percent, respectively) compared with Blacks (14 percent) (Caetano and Clark 2000). Among Hispanics, those born in the United States were approximately three times more likely to engage in drinking and driving than those who were born elsewhere. Hispanic men also had higher rates of ever having been arrested for driving under the influence of alcohol (19 percent), compared with White (13 percent) and Black (11 percent) men. Compared with other ethnic groups in Alaska, Alaska Natives had higher rates of car crashes and resulting injuries as well as arrests for driving while intoxicated (Segal 1998).

Research has also examined ethnic differences in alcohol-related violence between spouses or intimate partners. A national probability study conducted in 1995 revealed that the presence of alcohol-related problems, defined as either symptoms of alcohol dependence or drinking-related social consequences, was a strong predictor of intimate partner violence among Black couples, after controlling for the influence of sociodemographic factors, psychosocial variables, and level of alcohol consumption (Cunradi et al. 1999). Alcohol-related problems among both males and females were significant predictors of male-to-female partner violence as well as female-to-male partner violence among Blacks. However, the relationship between alcohol-related problems and intimate partner violence for Whites and Hispanics depended on the context. For example, alcohol-related problems among females were significant predictors of female-to-male partner violence for Whites but not for Hispanics.

In addition to the legal and social problems discussed thus far, alcohol use is also a factor in rates of death from various causes. Ethnic differences in deaths from alcohol-related cirrhosis and from car crashes are described below.

## Alcohol-Related Mortality

Alcohol abuse and dependence are associated with several medical problems. Cirrhosis mortality, however, usually serves as an indicator of the effects of heavy alcohol consumption in a population because heavy drinking is the most important risk factor for this disorder (Makimoto 1998).

For 1999, “chronic liver disease and cirrhosis” does not appear among the top 10 leading causes of death for Whites, Blacks, or Asians and Pacific Islanders (Centers for Disease Control and Prevention 2001). However, it was the sixth leading cause of death for both Hispanics and American Indians/Alaska Natives. Among Hispanics, the largest group of cirrhosis decedents was of Mexican background (Stinson et al. 2001). Compared with Whites, Hispanics have a higher prevalence of hepatitis C, a serious infectious liver disease that greatly increases the risk for liver damage in heavy drinkers (Lieber 2001). In 1997, Blacks had lower mortality rates for cirrhosis than Hispanics but higher rates than Whites (Stinson et al. 2001).

Among males ages 55 to 64 in each ethnic group, however, “chronic liver disease and cirrhosis” is one of the top 10 leading causes of death (Anderson 2001). The rates at which these disorders occurred in 1999, per 100,000 men in this age range were: 34.7 for Whites, 45.3 for Blacks, 100.6 for Native Americans, 11.5 for Asians and Pacific Islanders, and 61.8 for Hispanics (Anderson 2001). Blacks and Hispanics may have had higher rates of these disorders than Whites in part because both Blacks and Hispanics had higher rates of frequent heavy drinking than Whites, although their rates of drinking were lower than those of Whites (Caetano and Clark 1998*a*).

The rates of alcohol-related motor vehicle fatalities also vary among ethnic groups. Between 1990 and 1994, Whites and Blacks had similar rates of alcohol involvement in motor vehicle fatalities (44.2 percent and 45.2 percent, respectively) (National Highway Traffic Safety Administration [NHTSA] 1999). Among Hispanics, the proportion of motor vehicle fatalities that were alcohol related was 54.6 percent for Mexican Americans and 36.6 percent for Cuban Americans. Native Americans had the highest rate, at 68.1 percent, and Asians and Pacific Islanders had the lowest rate, at 28.2 percent (NHTSA 1999).

Thus far this paper has focused on how rates of consumption and alcohol-related problems and mortality vary for different ethnic groups. The next sections address the possible reasons for these variations.

## Explaining Ethnic Differences in Alcohol Use

Social and cultural factors as well as biological factors are thought to account for the differences in alcohol consumption and related problems among ethnic groups.

### Social and Cultural Factors

Alcohol use patterns can be influenced by social and cultural factors such as ethnic groups’ norms and attitudes regarding alcohol use and the extent of their acculturation to the larger U.S. society. A group’s norms refer to how one should behave in relation to alcohol—for example, beliefs about how much drinking is appropriate for a parent in the presence of small children, for a man at a bar with friends, or for someone at a party at another person’s home. A group’s attitudes refer to general beliefs about drinking, such as whether drinkers have more friends, whether a party is not really a party unless alcoholic beverages are served, and whether “getting drunk” occasionally is acceptable (Caetano and Clark 1999).

Alcohol norms and attitudes have been found to be strong predictors of drinking (Caetano and Clark 1999). People with more liberal alcohol norms and attitudes are more likely to be current drinkers than are those with more conservative norms and attitudes, based on 9 survey items reflecting whether respondents endorsed “any drinking” in various social settings and 11 items reflecting attitudes toward drinking and drunkenness. Similarly, people who have more liberal norms and attitudes are generally more likely to be frequent heavy drinkers than are those with more conservative beliefs. In national probability samples, both Blacks and Hispanics have reported more conservative alcohol norms and attitudes than Whites (Caetano and Clark 1999), which are reflected in the greater abstention rates (shown in table 1) among Blacks and Hispanics than among Whites. Hispanics also seem to have more extreme attitudes toward alcohol than Whites and Blacks (Caetano and Medina Mora 1990). In other words, their views are usually both positive and negative about the consequences, both good and bad, associated with drinking.

In addition to an ethnic group’s alcohol norms and attitudes, the degree to which a group is acculturated to the larger U.S. society also influences alcohol use among its members. Among Hispanic men, the drinking patterns of those who are more acculturated more closely resemble drinking patterns among the general U.S. population than those of less acculturated Hispanic men. Acculturation was based on 12 survey items measuring areas such as the daily use of and ability to speak, read, and write English and Spanish; a preference for media in English and Spanish; the ethnicity of the people with whom one interacts; and values thought to be characteristic of the Hispanic way of life (Caetano 1987). Among Hispanic women, acculturation is positively associated with drinking at all and with frequent “high maximum” drinking (drinking once a week, or more often, and having five or more drinks at a sitting at least once a year).

This association between acculturation and drinking patterns has also been found among other ethnic minority groups. Japanese Americans report drinking patterns that are more similar to those of Whites than to those of Japanese living in Japan (Higuchi et al. 1994). The proportion of Japanese men living in Japan who have problems associated with heavy drinking is higher among middle-aged drinkers (30–59 years old) and lower in both the youngest (18–29 years old) and oldest (60 years old and older) age groups. Among Japanese American men, the highest proportion of drinkers with heavy drinking problems is found among the youngest age group, as is also the case among White American men. This difference between Japanese men in Japan and Japanese American men likely reflects the influence of acculturation.

Acculturation to U.S. drinking patterns, however, may not be the same across different ethnic groups or even for all subcategories of nationalities within the same ethnic group (e.g., Japanese, Vietnamese, and Cambodians among Asian Americans). Differences in alcohol drinking patterns among U.S. Whites of various nationality backgrounds have been found to remain stable despite many generations of acculturation (Dawson 1998). Thus, Americans of Irish heritage report a higher frequency of heavy drinking than do White Americans in general, and those of English/Scottish, Austrian, Italian, and Greek backgrounds all report lower frequencies of heavy drinking than do White Americans in general.

One factor that contributes to how the association between acculturation and drinking patterns develops for a particular group or subgroup is the drinking patterns of that group’s country of origin. Hispanics from different Latin American countries, for example, bring a variety of drinking patterns when they migrate to the United States (Caetano 1987). Another factor contributing to the association is the particular place where a person settles. Different regions of the United States have their own drinking norms and customs, and people acculturate to different cultural environments (Caetano 1987). Thus, many factors influence the role that acculturation plays in the development of drinking patterns among ethnic minority groups (Caetano 1987).

### Biological Factors

Lower rates of alcohol use and alcoholism among Asians and Pacific Islanders appear to be related to a genetic variation prevalent in these populations (Makimoto 1998). Specifically, Asians are more likely than Whites to have a specific variant of a gene (i.e., an allele) called the aldehyde dehydrogenase-2 (ALDH2) Lys 487. This allele causes the body to break down alcohol in such a way that a person with the allele experiences symptoms such as facial flushing, nausea, headache, dizziness, and rapid heartbeat—collectively known as the flushing response—after consuming alcohol. Because of the presence of this allele, Asian populations tend to consume less alcohol and have lower levels of alcoholism than other ethnic groups (Makimoto 1998). This allele therefore may provide Asians some protection against heavy drinking and alcoholism (Yin et al. 1988; Makimoto 1998). The presence of the ALDH2 allele varies among Asian groups in the United States. In a study of college students, researchers found that 48 percent of students of Chinese ancestry had this allele, compared with 35 percent of students of Korean background (Luczak et al. 2001). The Chinese students also had a lower rate of binge drinking (7 percent) compared with the Korean students (30 percent).

### Other Proposed Factors

Other hypotheses have been advanced as explanations for the differences in drinking patterns among ethnic groups, but have not been supported by research findings. The first suggests that machismo, an exaggerated view of masculinity, plays an important role in the heavy drinking patterns of Mexican Americans. Close examination of machismo among White, Black, and Mexican American men, however, has shown that machismo is related to alcohol use among men irrespective of ethnic group and that it is not a valid explanation for the high levels of drinking among Mexican Americans (Caetano et al. 1998; Neff et al. 1991).

A second unsubstantiated hypothesis has attempted to explain the heavy drinking patterns of Native Americans by suggesting that they are biologically predisposed to heavy drinking and unable to control their drinking. The problems with this hypothesis are, first, that Native Americans do not appear to have a greater physiological or psychological reaction to alcohol than do members of other ethnic groups, and, second, that Native American groups in the United States vary greatly in their alcohol use (Caetano et al. 1998).

Single-variable explanations, such as these for Mexican Americans and American Indians, are often inadequate to explain the ethnic differences that exist in drinking patterns (Caetano et al. 1998).

## Alcoholism Treatment

In contrast with the amount of information available on the drinking patterns and alcohol-related problems of ethnic minority populations, research on alcoholism treatment with these groups is very limited (Caetano 1993). This is true in spite of the fact that both Hispanics (Arciniega et al. 1996; Caetano 1993) and Blacks (Kaskutas et al. 1999) are over-represented in treatment compared with their proportions in the general population. Information on alcoholism treatment is especially lacking for Asians/Pacific Islanders.

Nonetheless, the alcoholism treatment literature does reveal some ethnic differences. Kaskutas and colleagues (1999) found that a higher proportion of Blacks in treatment report having had some previous treatment compared with Whites. In addition, Blacks are more likely to have gone to inpatient programs and Whites to driving-under-the-influence programs. Blacks are almost twice as likely as Whites to have gone to Alcoholics Anonymous (AA) meetings as a part of their treatment. Blacks are also more likely than Whites to report having a spiritual awakening through being in AA and to have served at an AA meeting (e.g., by helping newcomers, setting up chairs, making coffee, cleaning up after a meeting). Whites are more likely than Blacks to report having a sponsor and reading AA literature.

Arroyo and colleagues (1998) found that Hispanics in treatment attend significantly more formal alcoholism therapy sessions and fewer AA meetings than Whites. Nonetheless, the different treatment options result in similar post-treatment drinking outcomes. The authors speculate that Hispanics may make greater use of their existing social support system and thus may not need AA as a support system, or that their treatment preferences may reflect a belief that AA is not as effective as formal treatment programs. The extent to which these findings may be generalized to other Hispanic populations may be limited by the fact that the Hispanics studied by Arroyo and colleagues (1998) were generally highly acculturated.

Limited research is available on the results of behavioral treatments and pharmacotherapy with Native Americans and Alaska Natives (Abbott 1998). Given the diversity of Native American and Alaska Native tribes, AA may be more appropriate for use by some tribes than others (Abbott 1998). The AA treatment that is used by these populations is often modified to meet their particular needs. In addition, given the many socioeconomic problems faced by many Native American and Alaska Native communities, such as high unemployment and low family incomes, Abbott suggests that focusing only on the alcohol-abusing segment of the population may be shortsighted. Efforts to address other community problems should at least parallel the development of appropriate alcoholism interventions. (See the recent research monograph published by the National Institute on Alcohol Abuse and Alcoholism [Mail et al. 2002] for more information on alcoholism treatment in this population.)

## Conclusion

The alcohol literature demonstrates that much variation exists in alcohol consumption, alcohol-related problems, and other issues among and within specific ethnic groups. An awareness of these differences can assist in identifying the subpopulations most at risk for developing alcohol-related problems.

Additional research is needed to address the gaps that currently exist in the literature. Many of the large national probability studies examining alcohol use focus primarily on Whites, Blacks, and Hispanics and do not include Asians/Pacific Islanders or Native Americans/Alaska Natives. Also, limited research exists on the alcohol consumption patterns of the newer immigrant populations in the United States. Alcohol use patterns for the longer-established Asian American populations of Chinese and Japanese may not reflect the patterns of the more recently settled populations of Southeast Asians such as Cambodians, Laotians, and Vietnamese (D’Avanzo 1997).

Further research is also needed on alcoholism treatment outcomes for ethnic minorities, including both general treatment interventions and those developed for particular minority groups. Information about ethnic group differences can be used to develop alcoholism treatment programs that are culturally specific or culturally sensitive (Caetano and Galvan 2001). Culturally specific programs are those developed for a particular ethnic group. Culturally sensitive programs incorporate elements from a given culture, such as language, cultural symbols, and so on, in programs that have been developed for the majority population. Presumably, interventions that are culturally specific are also culturally sensitive; however, interventions that are culturally sensitive need not be culturally specific. Both culturally specific and culturally sensitive programs are intended to be more acceptable to ethnic minority communities and hence more utilized by them.

To make alcoholism treatment more culturally sensitive for Hispanics, for example, it may be important to consider that the family plays a very important role in Hispanic culture, with family relationships bound by a strong sense of loyalty and reciprocity (Gaines et al. 1997; Lawrence et al. 1992). For this reason, alcoholism treatment programs for Hispanics may be improved by increasing family involvement.

Further research is needed to show whether culturally specific and culturally sensitive programs in fact yield better treatment outcomes than generic treatment programs, and whether treatment outcomes for these different types of programs vary by ethnic group. It is possible that long-established ethnic groups (such as Blacks) may be more likely to benefit from generic treatment programs than ethnic groups of more recent arrivals (such as the Hmong). It is also possible that the advantage of culturally specific or culturally sensitive programs is not so much based on their effectiveness but on the fact that they are better than generic programs at attracting and retaining ethnic minority clients.

Interventions with ethnic groups can also be developed to target the wider community, for example, by addressing the environmental factors that affect alcohol-related problems, such as the number of alcohol vending outlets in a particular neighborhood (Alaniz 1998). This type of effort may be appropriate in many communities with disproportionately high concentrations of alcohol vending outlets and alcohol advertising (Alaniz 1998).

Measures such as these—developed as a result of the work of alcohol researchers, treatment providers, and policymakers—can help decrease heavy drinking and alcohol-related problems among ethnic groups.

## Figures and Tables

**Table 1:** Alcohol Consumption Patterns Among People Age 18 and Older. Percentage of Whites, Blacks, and Hispanics Who Abstained From Alcohol or Were Frequent Heavy Drinkers, by Gender, 1984 and 1995

	Abstention From Alcohol		Frequent Heavy Drinking*	

	1984 %	1995 %	1984 %	1995 %
**Men**				
White	24	26	20	12
Black	29	36	15	15
Hispanic	22	35	17	18
**Women**				
White	34	39	5	2
Black	46	55	5	5
Hispanic	47	57	2	3

* Frequent heavy drinking is defined as drinking five or more drinks at a sitting at least once a week.

SOURCE: Caetano and Clark 1998*a*.

**Table 2:** Prevalence of Alcohol-Related Problems Among People Age 18 and Older. Percentage of Whites, Blacks, and Hispanics Who Reported Zero, One, Two, or Three or More Alcohol-Related Problems

Number of Alcohol-Related Problems	Whites		Blacks		Hispanics	

	1984 %	1995 %	1984 %	1995 %	1984 %	1995 %
**Men**						
0	75	78	73	75	83	71
1	8	7	7	9	5	8
2	5	4	4	3	4	5
3+	12	11	16	13	9	16
**Women**						
0	86	88	90	89	94	91
1	6	5	5	4	2	3
2	3	3	2	2	1	1
3+	5	4	3	4	3	5

SOURCE: Caetano and Clark 1998*b*.

## Footnotes

1 Population groups other than Whites are considered racial/ethnic minorities for the purposes of this article.

2 A standard drink is defined as 12 ounces of beer, 5 ounces of wine, or 1.5 ounces of 80-proof distilled spirits, all of which contain the same amount of alcohol (U.S. Department of Health and Human Services and U.S. Department of Agriculture 1995).

3 Other national surveys have reported less dramatic differences in heavy drinking rates among ethnic groups than those reported in this article. For example, estimates of heavy drinking from the 2000 National Household Survey on Drug Abuse for people age 18 or older (data for men and women combined) are 6.6 percent for Whites, 4.5 percent for Blacks, and 4.7 percent for Hispanics (Substance Abuse and Mental Health Services Administration 2001). In this survey, heavy drinking was defined as drinking five or more drinks on the same occasion on 5 or more days in the past 30 days.
